# Supplementary material for: Spontaneous Neutrophil Extracellular Traps Release Are Inflammatory Markers Associated with Hyperglycemia and Renal Failure on Diabetic Retinopathy
Source: Biomedicines. 2023 Jun 22;11(7):1791. doi: 10.3390/biomedicines11071791 (PMC10376331; doi:10.3390/biomedicines11071791)
Supplement: Supplementary file 1 [file biomedicines-11-01791-s001.zip › biomedicines-2450088-supplementary.pdf]

Table S1. Demographic data and clinical evaluation of recruited subjects.

Participants (n=89)	without DM-2 (n=15)	DM-2 without RD (n=15)	DM-2 DRNPMi (n=15)	DM-2 RDNP M (n=15)	DM-2 RDNPS (n=14)	DM-2 DRP (n=15)	*p* value
Personal history							
**Age (Years)**	39.33[(a)*] (±10.85)	52.27 (±9.02)	55.27 (±12.46)	62.47 (±9.75)	61.86 (±8.43)	59.00 (±10.88)	$*p< 0.05$
**Sex (M/F)**	7/8	7/8	4/11	5/10	7/7	9/6	
Non pathological							
**Proper diet (Y/N)**	9/6	7/8	9/6	11/4	11/3	2/13	
**Smoker (Y/N)**	3/12	3/12	12/3	1/14	4/11	12/3	
Pathological history							
**Diagnosis of DM-2 (Years)**	N/A	7.20[(b)*] (±4.37)	13.07 (±5.95)	17.93 (±5.36)	17.64 (±3.22)	22.13 (±5.76)	$*p< 0.05$
**BMI**	25.67[(c)*] (±3.84)	30.59 (±4.63)	28.02 (±2.25)	27.68 (±4.00)	28.45 (±5.62)	28.00 (±4.14)	$*p< 0.05$
**Hypertension (Y/N)**	1/14	8/7	4/11	13/2	5/7	10/5	
**Mean arterial pressure (mmHg)**	90.00 (±6.32)	91.29 (±15.17)	86.09 (±24.87)	96.04 (±11.24)	96.12 (±10.28)	96.71 (±9.62)	
**eGFR (mL/min/1.73m^2)**	112.5[(d)*] (±15.92)	107.2 (±18.09)	104.6 (±29.12)	72.74 (±36.09)	77.59 (±28.93)	74.80 (±24.15)	$*p< 0.05$
**Iron (µg/dL)**	105.8 (±40.97)	86.67 (±27.82)	82.80 (±19.61)	79.00 (±31.20)	76.91 (±28.64)	75.09 (±27.06)	
Drug use							
Insulin (Y/N)	0/15	4/11	7/7	6/9	7/7	8/7	
Other hypoglycemic agents (Y/N)	0/15	7/8	3/11	4/10	4/10	5/10	

Red series hematic biometry							
Erythrocytes ($10^6/\mu L$)	5.219 (±0.60)	5.119 (±0.55)	5.049 (±0.41)	4.857 (±0.52)	4.922 (±0.90)	5.231 (±0.40)	
Hemoglobin (mg/dL)	14.89 (±1.74)	14.20 (±1.37)	14.47 (±1.19)	13.27 (±2.02)	13.66 (±2.37)	14.55 (±1.34)	
Hematocrit (%)	45.49 (±4.57)	43.67 (±3.21)	43.79 (±2.64)	41.80 (±4.28)	45.96 (±13.71)	44.83 (±3.41)	
MCV (fL)	87.06 (±3.30)	86.10 (±9.43)	86.85 (±3.61)	85.83 (±3.22)	84.23 (±8.77)	85.81 (±3.48)	
White series and platelets							
Platelets ($10^3/\mu L$)	257.1 (±57.12)	234.9 (±68.23)	225.1 (±64.02)	225.6 (±55.12)	256.9 (±86.08)	219.6 (±58.59)	
Total leukos ($10^3/\mu L$)	6.321 (±2.13)	5.987 (±1.61)	5.491 (±1.26)	6.116 (±1.38)	6.077 (±1.26)	6.063 (±1.18)	
Total neutrophils ($10^3/\mu L$)	4.13 (±1.78)	3.65 (±1.43)	3.29 (±0.81)	3.91 (±1.26)	3.79 (±1.08)	3.74 (±0.94)	
Basophils ($10^3/\mu L$)	0.014 (±0.009)	0.020 (±0.025)	0.018 (±0.011)	0.014 (±0.013)	0.015 (±0.013)	0.019 (±0.007)	
Eosinophils ($10^3/\mu L$)	0.19 (±0.32)	0.13 (±0.10)	0.15 (±0.15)	0.26 (±0.32)	0.12 (±0.08)	0.16 (±0.08)	
Lymphocytes ($10^3/\mu L$)	1.64 (±0.61)	1.81 (±0.40)	1.66 (±0.54)	1.63 (±0.43)	1.66 (±0.48)	1.71 (±0.51)	
Clinical Biochemistry							
HbA1c (%)	5.38**[(a)*]** (±0.18)	7.92 (±2.03)	9.32 (±2.37)	8.96 (±2.68)	8.27 (±2.11)	8.44 (±1.74)	*$p < 0.05$
Fasting glucose (mg/dL)	88.67**[(a)*]** (±6.86)	167.20 (±65.41)	184.70 (±82.87)	187.10 (±84.98)	167.44 (±78.99)	145.30 (±51.58)	*$p < 0.05$
Urea (mg/dL)	25.47**[(d)*]** (±5.19)	29.40 (±5.34)	32.27 (±9.31)	50.33 (±20.21)	43.86 (±26.86)	43.40 (±14.21)	*$p < 0.05$
Creatinine (mg/dL)	0.75 (±0.13)	0.73 (±0.13)	0.70 (±0.22)	1.11 (±0.56)	1.44 (±2.06)	1.05 (±0.33)	

Total cholesterol (mg/dL)	181.9 (±29.72)	185.1 (±48.21)	189.8 (±69.56)	190.1 (±63.31)	175.5 (±51.98)	173.1 (±37.56)	
LDL cholesterol (mg/dL)	119.9 (±25.18)	124.7 (±44.52)	118.7 (±61.92)	125.3 (±57.44)	111.5 (±36.55)	105.9 (±41.18)	
Triglycerides (mg/dL)	124.0 (±36.98)	190.7 (±78.97)	168.1 (±75.21)	130.1 (±48.20)	161.9 (±93.73)	246.5 (±249.7)	
Uric acid (mg/dL)	5.63 (±1.62)	5.16 (±1.25)	4.63 (±1.35)	5.71 (±2.18)	5.92 (±1.51)	6.58 (±1.46)	
Total proteins (g/dL)	7.34 (±0.42)	7.26 (±0.40)	7.06 (±0.65)	7.00 (±0.41)	6.98 (±0.59)	6.71 (±1.65)	
Albumin (mg/dL)	4.64 (±0.29)	4.46 (±0.21)	4.38 (±0.35)	4.18 (±0.43)	4.30 (±0.43)	4.34 (±0.33)	
Globulin (mg/dL)	2.70 (±0.34)	2.79 (±0.35)	2.62 (±0.43)	2.82 (±0.37)	2.68 (±0.45)	2.77 (±0.37)	
AST (U/L)	19.67 (±5.31)	22.00 (±8.85)	22.60 (±4.33)	20.53 (±5.44)	24.50 (±8.59)	20.89 (±13.53)	
ALT (U/L)	22.60 (±8.90)	30.47 (±15.71)	24.47 (±6.66)	21.93 (±11.94)	26.64 (±12.72)	21.06 (±11.09)	
LDH (U/L)	165.7 (±22.37)	153.4 (±26.47)	182.9 (±36.13)	193.1 (±26.91)	184.2 (±52.67)	180.3 (±30.72)	
GGT (U/L)	27.00 (±12.94)	51.40 (±52.20)	31.53 (±21.54)	31.80 (±30.88)	42.71 (±34.35)	27.80 (±16.70)	
Alkaline phosphatase (U/L)	82.13 (±20.14)	99.27 (±37.03)	118.30 (±42.42)	103.10 (±33.01)	108.20 (±26.72)	87.13 (±14.87)	
Amylase (U/L)	71.42 (±17.95)	69.92 (±18.26)	65.55 (±19.75)	68.67 (±26.99)	81.08 (±27.83)	83.75 (±28.57)	
Uroanalysis							
Density	1.026[(e)]* (±0.004)	1.021 (±0.008)	1.017 (±0.005)	1.016 (±0.007)	1.016 (±0.006)	1.019 (±0.006)	*$p < 0.05$
pH	5.929 (±0.70)	5.433 (±0.56)	5.767 (±0.72)	5.867 (±0.54)	6.154 (±0.77)	6.100 (±0.84)	
Glucose (mg/dL)	0.0	216.7 (±329.3)	283.3 (±315.5)	263.3 (±303.8)	238.5 (±384.1)	40.0 (±129.8)	

**Proteins (mg/dL)**	**2.00 (±7.74)**	**0.00**	**26.00 (±19.83)**	**62.00 (±21.07)**	**3.84 (±21.07)**	**35.33 (±20.95)**

Abbreviations: (Y/N), yes/no; BMI, body mass index; GFR, glomerular Filtration Ratio; MCV, mean corpuscular volume; HbA1c, glycated hemoglobin; LDL, low density lipoproteins; AST, Aspartate Aminotransferase; ALT, Alanine Aminotransferase; LDH, Lactate Dehydrogenase; GGT, gamma-glutamyl transferase. *$p<0.05$ [a) Without DM-2 group v.s. all groups; (b) DM-2 without RD group v.s. all groups; (c) Without DM-2 group v.s. DM-2 without DR; d) Without DM-2 group v.s. DM-2 RDNPM, DM-2 RDNPS and DM-2 DRP; (e) Without DM-2 group v.s. DM-2 DRNPMi, DM-2 RDNPM, DM-2 RDNPS and DM-2 DRP]. **Proper diet**: refers a balanced diet with the consume of all nutriments and appropriate caloric portions according with weight, tall and age of individuals. Each meal at least must include three different groups of nutriments such as vegetables and fruits (1), cereals and tubers (2), legumes and animal products (3). Do not consume or low consumption of sugary foods and drinks, as well as fried foods. **Obesity:** condition with a BMI upper at 30. **Hypertension:** considered as a systolic pressure equal or upper at 140 mmHg, and a diastolic pressure equal or upper at 90 mmHg. **Glucose control:** considered as a fasting glucose of ≤130 mg/dL and HbA1c ≤ 7%.
